# Karyotypic assignment of Sri Lankan *Anopheles culicifacies* species B and E does not correlate with cytochrome oxidase subunit I and microsatellite genotypes

**DOI:** 10.1186/s13071-015-0944-9

**Published:** 2015-06-14

**Authors:** Sinnathamby N. Surendran, Nathan Truelove, Devojit K. Sarma, Pavilupillai J. Jude, Ranjan Ramasamy, Kanapathy Gajapathy, Lalanthika B. S. Peiris, S. H. P. Parakrama Karunaratne, Catherine Walton

**Affiliations:** Department of Zoology, Faculty of Science, University of Jaffna, Jaffna, 40000 Sri Lanka; Faculty of Life Sciences, University of Manchester, Oxford Road, Manchester, M13 9PT UK; Regional Medical Research Centre, NE region (ICMR), Dibrugarh, 786001 Assam India; Faculty of Science and Technology, Anglia Ruskin University, Cambridge, CB1 1PT UK; Regional Office, Anti Malaria Campaign, Hambantota, 82000 Sri Lanka; Department of Zoology, Faculty of Science, University of Peradeniya, Peradeniya, 20400 Sri Lanka

**Keywords:** *Anopheles culicifacies*, *COI*, Malaria, Microsatellite, Mosquito vector, Species complex, Sri Lanka, Y-chromosome karyotype

## Abstract

**Background:**

The identification of species B and E in the *Anopheles culicifacies* complex in the Indian subcontinent has been based on Y-chromosome karyotype. Since no detectable variations were previously found in DNA markers commonly used for sibling species identification, further molecular characterization using cytochrome oxidase subunit I (*COI*) and microsatellite markers was carried out on Y-chromosome karyotyped *Anopheles culicifacies* specie B and E from Unnichchai, Kallady and Ranawarunawa in Sri Lanka.

**Findings:**

*COI* sequence analysis (n = 22) revealed the presence of nine unique haplotypes with six in each species. Three haplotypes were shared by both species. The two sibling species had a pairwise F_ST_ value of 1.338 (p < 0.05) with the number of migrants (Nm) value <1. The genetic structure analysis resulted in two genetic clusters not 100 % associated with karyotypes. While none of the species B were incorrectly assigned two were inconclusive. Five out of 26 specimens karyotyped as species E were incorrectly assigned, while further 9 were inconclusive.

**Conclusions:**

The new molecular data support the existence of two genetically different populations of the Culicifacies Complex in Sri Lanka that are not associated with the Y-chromosome karyotype. Detailed analysis with more microsatellite markers and assortative mating experiments are needed to establish the presence of the two genetically distinct populations and relate them to Y-chromosome morphology.

## Background

**Fig. 1:**
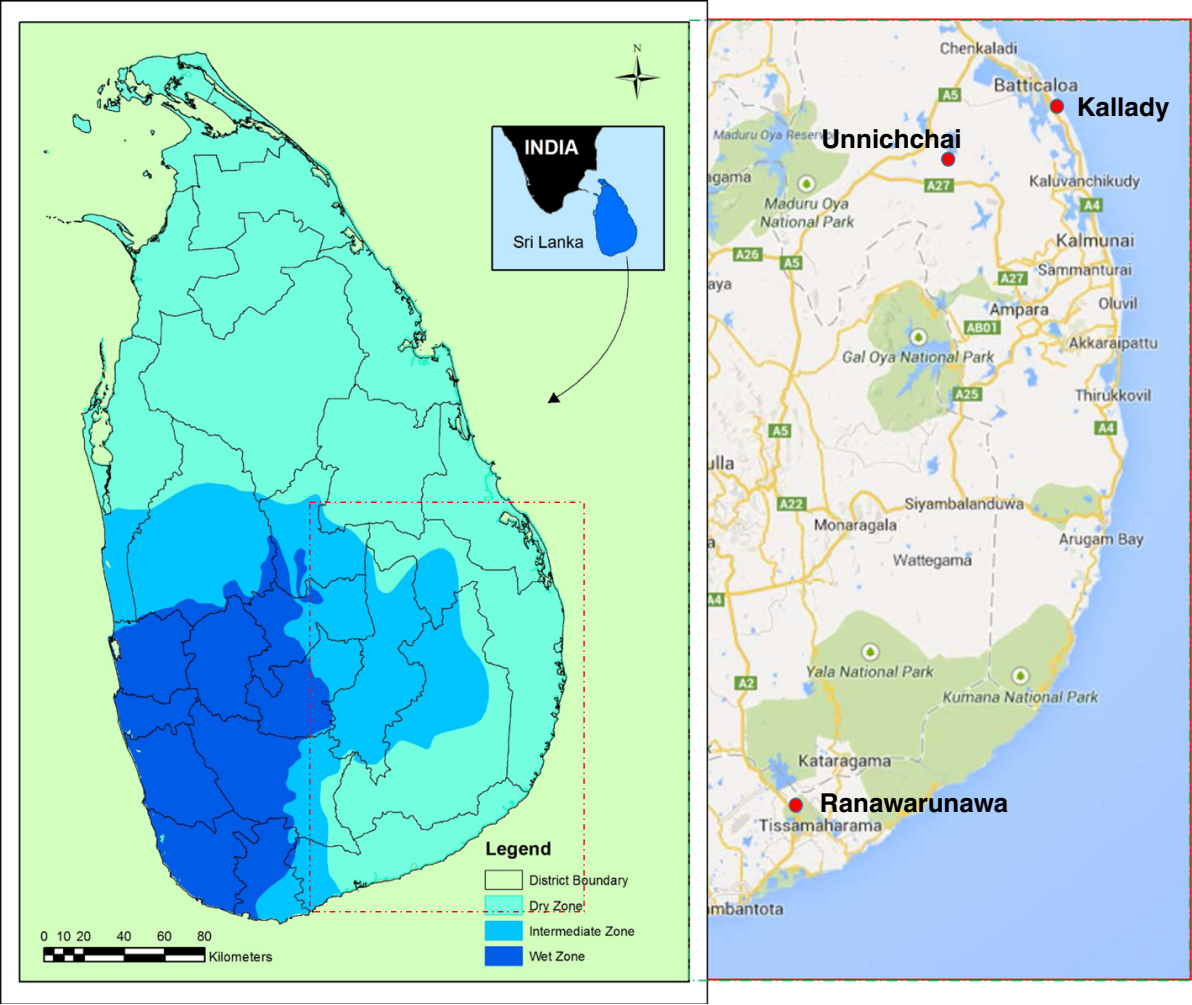
Mosquito collection sites in Sri Lanka. Map showing the three mosquito collection sites in Sri Lanka, its administrative district boundaries and rainfall zones and the proximity to South India

*Anopheles culicifacies* Giles sensu lato is the principal vector of malaria in Sri Lanka [1] and a dominant vector elsewhere in Asia [2]. The *Anopheles culicifacies* species complex in India is comprised of five sibling species A-E [2]. Sibling species E was recently described based on the relationship between Y-chromosome polymorphisms of male offspring and sporozoite infection of mothers in Rameshwaram Island in South India which is in close proximity to Sri Lanka (Fig. 1) [3]. In India, *An. culicifacies* sp. B and *An. culicifacies* sp. E share the same polytene chromosome banding patterns but differ in the position of centromeres in mitotic Y-chromosomes. The latter is acrocentric in species B and submetacentric in species E [3].

Early polytene chromosome mapping of Sri Lankan *An. culicifacies* suggested that only species B was present in Sri Lanka [4]. The conundrum that species B is a poor vector of malaria in India [2, 3] but the major vector in Sri Lanka was later resolved through Y-chromosome karyotyping Sri Lankan *An. culicifacies* populations in relation to their vectorial capacity [5]. This indicated that *An. culicifacies* in Sri Lanka was a mixture of the two putative sibling species B and E based on the presence of the two Y-chromosome karyotypes previously observed in India [5]. Further studies using karyotypically-identified mosquitoes showed that Sri Lankan species E differed from species B in being infectible with *Plasmodium falciparum* and *P. vivax* and possessing greater resistance to common insecticides [6].

Molecular characterization of the internal transcribed spacer 2 (ITS2) and D3 region of the ribosomal DNA (rDNA), cytochrome oxidase subunit II (*COII*) and the guanylate cyclase intron failed to differentiate Sri Lankan *An. culicifacies* sp. B and *An. culicifacies* sp. E identified by Y -chromosome karyotyping [7]. The present study was therefore carried out to further elucidate the species composition of *An. culicifacies* s.l. in Sri Lanka using the cytochrome oxidase subunit I (*COI*) gene, microsatellites and Y-chromosome karyotypes. Specimens with the two different Y-chromosome karyotypes in Sri Lanka are referred to as putative species in this article.

## Methods

### Study sites, sample collection and sibling species identification

Blood-fed anopheline mosquitoes were collected from February 2011 to July 2012 using cattle baited hut collections (CBHC) from three locations found in dry zone of Sri Lanka *viz.* Unnichchai, Kallady and Ranawarunawa (Fig. 1). Collected mosquitoes were identified at the species level with available morphological key [8]. Blood-fed *An. culicifacies* females were maintained individually and single female F_1_ progenies were raised as reported previously [5]. Late third and early fourth instar male larvae were used to karyotype Y-chromosomes to identify putative sibling species: acrocentric as species B and submetacentric as species E as described previously [5]. Karyotyped individuals from different F_1_ progenies were used for DNA-based characterisation.

### DNA extraction and amplification

DNA from karyotyped specimens of putative species B and E was extracted using phenol-chloroform [9]. A portion of the *CO1* gene present in mitochondrial DNA was amplified with primers C1-J-1718 and C1-N-2191 [10] as previously reported [9]. The PCR products were purified with the GenElute^TM^ PCR Clean-UP Kit (Sigma-Aldrich, USA). Purified PCR products were sequenced in both directions using the Big Dye Terminator V3.1 Cycle Sequencing Kit (Applied Biosystems, USA) on an ABI 3730 automatic DNA sequencer (Applied Biosystems, USA) at the University of Manchester core sequencing facility. Sequence chromatograms were edited manually in Geneious 4.8.5 [11] and compared with sequences available from GenBank using BLASTn.

### DNA sequence analysis and population genetic structure based on *COI* sequences

Genetic information e.g. number of haplotypes, segregating sites, haplotype diversity and nucleotide diversity was obtained using DnaSP 5.10 [12]. Analysis of molecular variance (AMOVA) [13] and estimation of pairwise F_ST_ values for chromosomal forms (species B and E) was performed in Arlequin 3.1 [14] and their significance tested by 1,000 permutations. A statistical parsimony based haplotype network for species B and E populations was created using TCS v1.21 [15].

### Microsatellite multiplex, fragment analysis and genotyping

Thirty nine unrelated individuals from the 3 populations were characterised by the Y-chromosome karyotype and used in the study. Five microsatellite markers capable of amplification from *An. culicifacies* sp. A and sp. B from India [16] were selected for this study *viz.* AcAIIB5, ACAVB93, AcAVIB213, AcA 36 and AcA59. Fluorescent-labelled (6-FAM®, NED®, VIC® and PET®) forward primers (Applied Biosystems, UK) and non-labelled reverse primers (Sigma-Aldrich) were used in a multiplex PCR reaction. Each individual reaction of 5 μl consisted of 0.5 μl of the 10x primer mix (each primer at 2 μM), 2.5 μl of Type-it Multiplex PCR Master Mix (QIAGEN), 1 μl of MQ water and 1 μl of genomic DNA (10–20 ng). The amplification conditions were; initial denaturation at 95 °C for 5 min, followed by 26 cycles of 95 °C for 30 s, 57 °C for 120 s, and 72 °C for 30 s. This was followed by final extension at 60 °C for 30 min. The PCR product was diluted with 5 μl MQ water and 0.5 μl of this was mixed with 9.5 μl of a mix consisting Hi-Di Formamide® (Applied Biosystem) and GeneScan – 500 LIZ Size Standard (37:1) prior to genotyping on an ABI 3730 automatic DNA sequencer using GeneMapper® v.3.7software (Applied Biosystems, USA).

### Microsatellite data analysis

Genetic structure of *An. culicifacies* s.l*.* was examined as a single population and two chromosomal forms using the program STRUCTURE version 2.3.4 [17], with a burn in of 100,000 for each value of K from 1 to 5. In order to quantify the amount of variation of the likelihood for each K, a data set of 20 runs were carried out. The appropriate K value was determined using STRUCTURE HARVEST online [18]. The effective migration rate (Nm) between the putative species was estimated using GENEPOP version 4.2 [19].

## Findings

Thirty nine isofemale larval progenies were karyotyped and 13 were identified putative *An. culicifacies* sp B and 26 as sp E. All mosquitoes collected from Unnichchai were identified as *An. culicifacies* sp. B and all from Kallady and Ranawarunawa as *An. culicifacies* sp. E.

### Genetic structure inferred from *COI sequences*

**Fig. 2:**
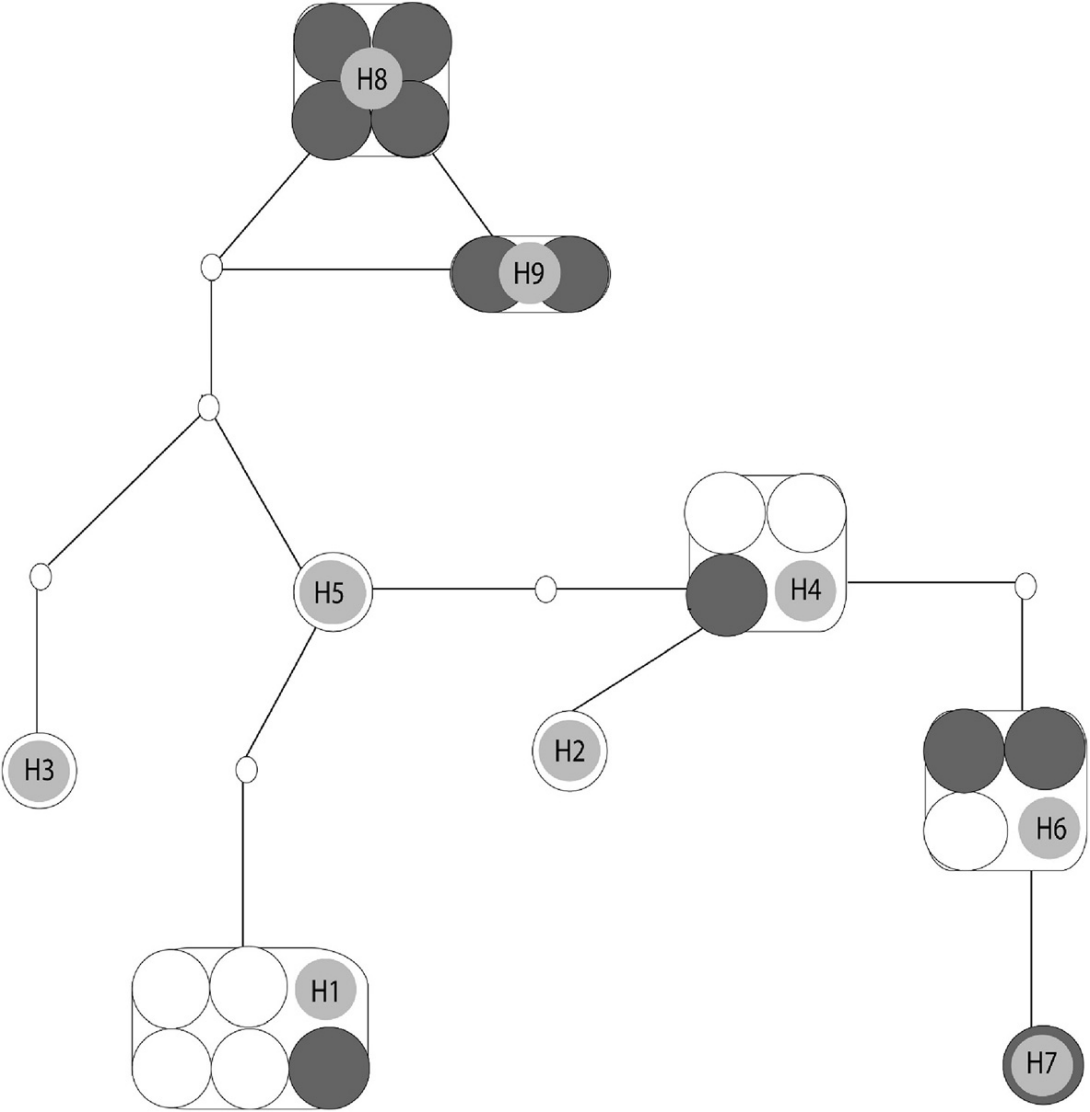
Haplotype network of *COI* of the *An. culicifacies* s.l. populations. The network is composed of sibling species B (black large circles) and E (white large circles). Large circles indicate individual sequences and the haplotype numbers are shown as H series. Small empty circles represent missing hypothetical haplotypes

Twelve putative species B and 10 putative species E were initially amplified and sequenced for *COI*. After trimming sequences to the same length, a dataset 449 bp in length, was used for analysis. The two putative species had a pairwise F_ST_ value of 0.1338 (*p* < 0.05). However the F_ST_ value of 0.139 (*p* < 0.05) found to be greater between the geographically more distant *An. culicifacies* sp. B of Unnichchai and *An. culicifacies* sp. E of Ranawarunawa. There were nine haplotypes altogether (the corresponding haplotype sequences are deposited in GenBank accession numbers KJ 010890 – KJ 010898) and the statistical parsimony haplotype network (Fig. 2) shows that three of these haplotypes (H1, H4 and H6) were shared by both putative species without any clustering associated with chromosomal forms.

### Genetic structure inferred from microsatellites

**Fig. 3:**
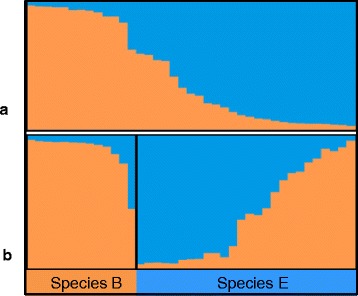
Clustering of putative species B and E of the Culicifacies Complex in Sri Lanka by STRUCTURE. The figure shows the assignment of individuals when they were analysed as (**a**) single population and (**b**) as putative species B and E based on chromosomal forms

Genetic structure analysis supported by STRUCTURE clustering algorithm analysis followed by STRUCTURE HARVEST revealed two genetic clusters in all analysis *viz*. as single population and two chromosomal forms (Fig. 3). When considered as a single population, the samples clearly grouped into two clusters (Fig. 3a) while as two chromosomal forms, although the samples were clustered into two, 11 out of 13 chromosomal form B and 12 out of 26 chromosomal form E were assigned correctly. While none of the *An. culicifacies* sp. B were incorrectly assigned two were inconclusive. In *An. culicifacies* sp. E, five out of 26 were incorrectly assigned while nine were inconclusive (Table 1; Fig. 3b). The number of migrants (Nm) value was <1 (0.55242).

**Table 1:** Assignment of karyotypically identified species B and E of the *Anopheles culicifacies* Complex based on microsatellites

Species assignment by karyotype	Number of individuals not assigned clearly	Number of individuals assigned to sp. B (q > 0.8)	Number of individuals assigned to sp. E (p > 0.8)
B	2	11	0
E	9	5	12

## Discussion

This is the first attempt to use microsatellite markers to characterize *An. culicifacies* s.l. populations in Sri Lanka. The *COI* sequence analysis of the two chromosomal forms resulted in three shared haplotypes suggests that the two putative species *An. culicifacies* sp. B and *An. culicifacies* sp. E in Sri Lanka are not reproductively isolated. However, the F_ST_ value of 0.1338 (*p* < 0.05) may be an indication that the populations of putative *An. culicifacies* sp. B and *An. culicifacies* sp. E have genetic differences. Because the distance between Unnichchai and Ranawarunawa is ~ 165 km, a somewhat higher F_ST_ value for *An. culicifacies* sp. B of Unnichchai and *An. culicifacies* sp. E of Ranawarunawa of 0.139 (*p* < 0.05), is an indication that geographical distance and barriers within Sri Lanka may have a role in genetic variation. Geographical influence is also supported by the presence of only one of the putative species at each of the three locations *viz. An. culicifacies* sp. B in Unnichchai and *An. culicifacies* sp. E in Kallady and Ranawarunawa. The observed low Nm value (<1) is however consistent with there being gene flow between the two putative sibling species.

The microsatellite analysis revealed the presence of two genetically distinct populations within the Culicifacies Complex in Sri Lanka but that these taxa are not delineated by Y-chromosome dimorphism. Of 39 karyotyped individuals, 11 of both *An. culicifacies* sp. B and *An. culicifacies* sp. E were inconclusive while 5 individuals of *An. culicifacies* sp. E were incorrectly assigned. This indicates that the detected population structuring is not associated with Y-chromosome assignment to putative species B and E.

It was suggested based on a study that correlated genetic structure of chromosomal forms of shrews (*Sorex araneus*) based on microsatellite data that karyotypic differences played a minor role in structuring the population relative to others such as geographic or historical factors [20]. Structural variations in the sex chromosomes are not associated with reproductive isolation [21]. The existence of *An. culicifacies* sp. E in India was based solely on Y-chromosome dimorphism associating with vector potentiality. Since the mothers of acrocentric Y-chromosome progeny were not infected with malaria parasites, they were designated as *An. culicifacies* sp. B and the infected mothers of males with metacentric Y-chromosomes as *An. culicifacies* sp. E [3]. The observed Y-chromosome dimorphisms [5] associated with differential vector potentiality [6] suggested that the Culicifacies Complex in Sri Lanka too is composed of two analogous sibling species *viz. An. culicifacies* sp. B (non vector) and *An. culicifacies* sp. E (vector), although assortative mating could not be tested.

At present no single technique is available to directly identify all five sibling species in the Culicifacies Complex in the Indian subcontinent. A recent study carried out in India confirmed the previous report on the lack of molecular differentiation between putative sibling species B and E of the Culicifacies Complex in Sri Lanka [7] and revealed that a mtDNA-*COII* based diagnostic assay [22] that was earlier reported to distinguish all five sibling species (A-E) in the Culicifacies Complex could not be used universally to distinguish all five members including *An. culicifacies* sp. B and *An. culicifacies* sp. E [23]. This study further suggests that the Culicifacies Complex is composed of only two distinct species *An. culicifacies* sp. A and *An. culicifacies* sp. B from which other members of Culicifacies Complex (species C, D and E) have recently diverged but not yet become reproductively isolated [23].

All the presently available evidence including phenotypic differences (e.g. infectivity with malaria parasites and insecticide resistance) and molecular data are compatible with a suggestion that divergence of *An. culicifacies* sp. B and *An. culicifacies* sp. E is a very recent event that may be part of ongoing speciation process with little molecular difference between the two forms. Therefore it is important to examine additional microsatellite markers and the possibility that acrocentricity or metacentricity in the Y-chromosome karyotype of *An. culicifacies* s.l., as commonly determined in the laboratory, may not be a robust marker for differentiating putative sibling species *An. culicifacies* sp. B from *An. culicifacies* sp. E (or non-malaria vector from malaria vector respectively) in Sri Lanka.

The presence of Y-chromosome dimorphism in vector sibling *An. culicifacies* sp. C of the Culicifacies Complex in India has already been reported [24]. However association of these two karyotypes with parasite transmission or their possible status as two sibling species is presently unknown.

Reproductive isolation in *An. gambiae* s. s. populations correlates with molecular variations rather than chromosomal differences [25]. It is possible that *An. culicifcaies* s.l. in Sri Lanka is composed of different populations with different biological and genetic properties but not wholly reproductively isolated. It is important to analyze *An. culicifacies* s.l. samples from other locations to establish an integral population genetic structure for Sri Lanka and compare this with that of South Indian *An. culicifacies*. Experiments to assess the capacity for mating and production of viable offspring between the two putative species identified through the two Y-chromosome karyotypes in Sri Lanka and in India are also necessary.
